# Vesico-Vaginal Fistula

**Published:** 1863-08

**Authors:** T. Spencer Wells


Vesico-Vaginal Fistula.—In operating for the cure of vesico-vaginal fistula, it will be found that fine strong silk answers even better than wire. It is passed much more easily and with finer needles, causes less irritation of the neighboring parts, and is removed much more easily, while union takes place quite as well as when wire is used. The use of the catheter may be dispensed with altogether. It is a most troublesome part of the after treatment, and, "I believe," it need not even be used to empty the bladder; moreover, the use of the catheter is not of itself quite free from danger, sometimes producing a species of catarrh of the bladder, persisting for months after the cure of the fistula.—(Mr. T. Spencer Wells.)—*Id.*

